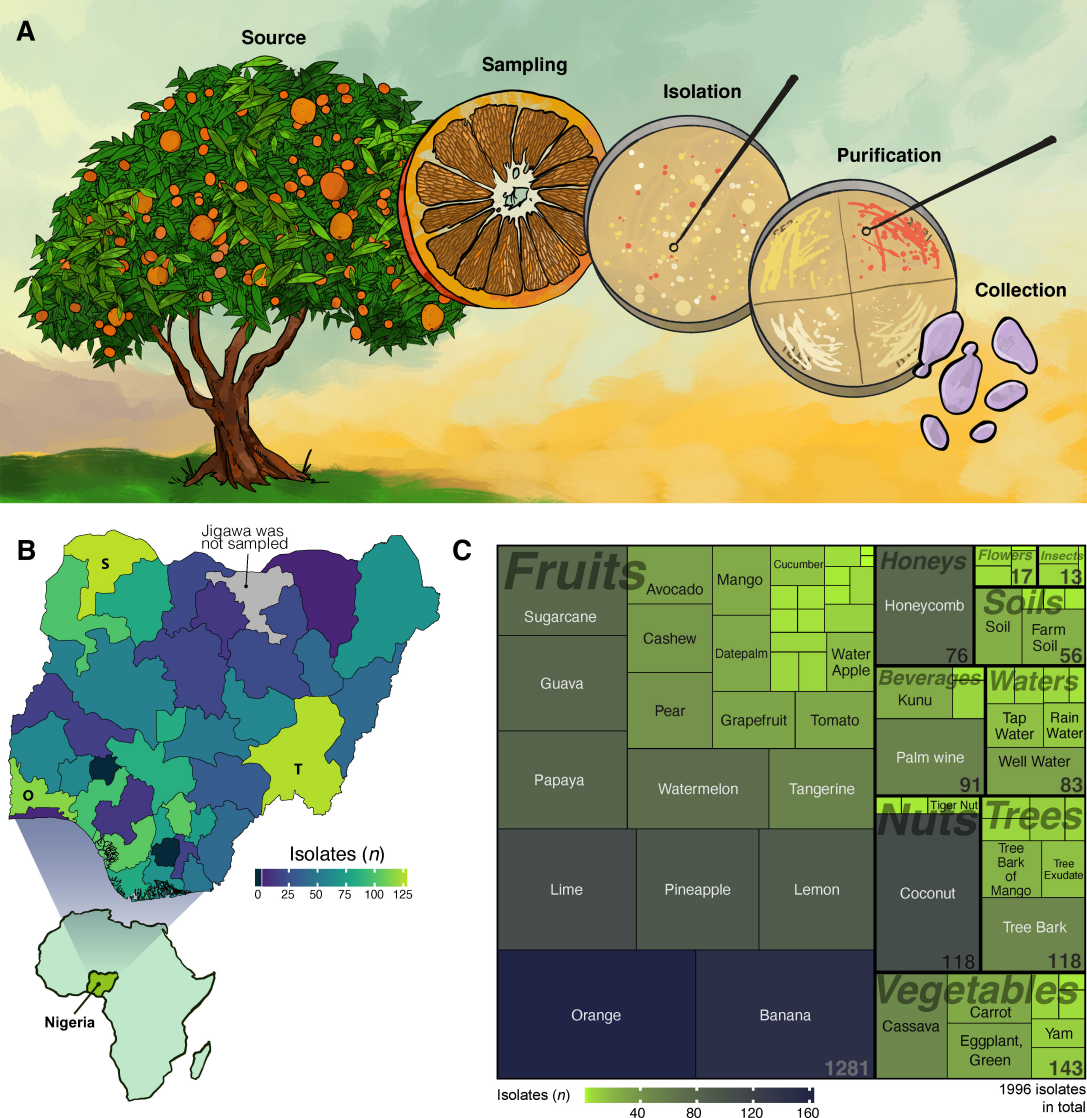# Articles of Significant Interest in This Issue

**DOI:** 10.1128/aem.00089-25

**Published:** 2025-01-31

## POSTDOC MATTERS THAT MATTER

Sabree et al. (e01483-24) discuss the financial stress that postdocs experience when relocating without institutional compensation for moving expenses. As the authors state in their article, “Solving this short-term liquidity pressure can increase productivity, job satisfaction, and the likelihood [postdocs] remain in academia.”


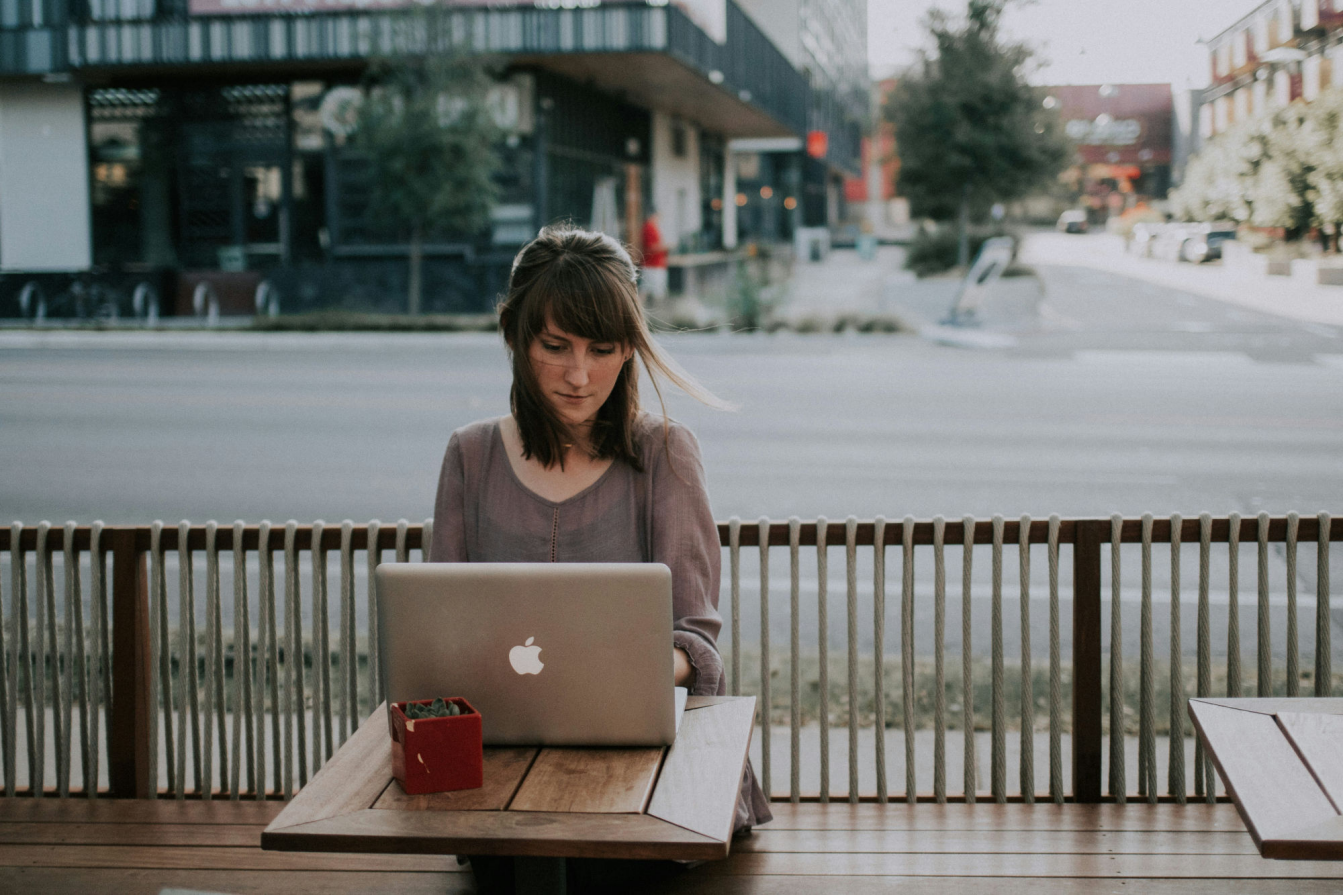


## A GENETIC VIEW OF THE METHANOTROPHIC S-LAYER

The genetic characterization of the intricate S-layer of methanotrophs by Hamilton et al. (e01364-24) suggests roles in cell envelope stability and metal scavenging as well as a biosynthetic route for the synthesis of self-assembling protein materials from methane.
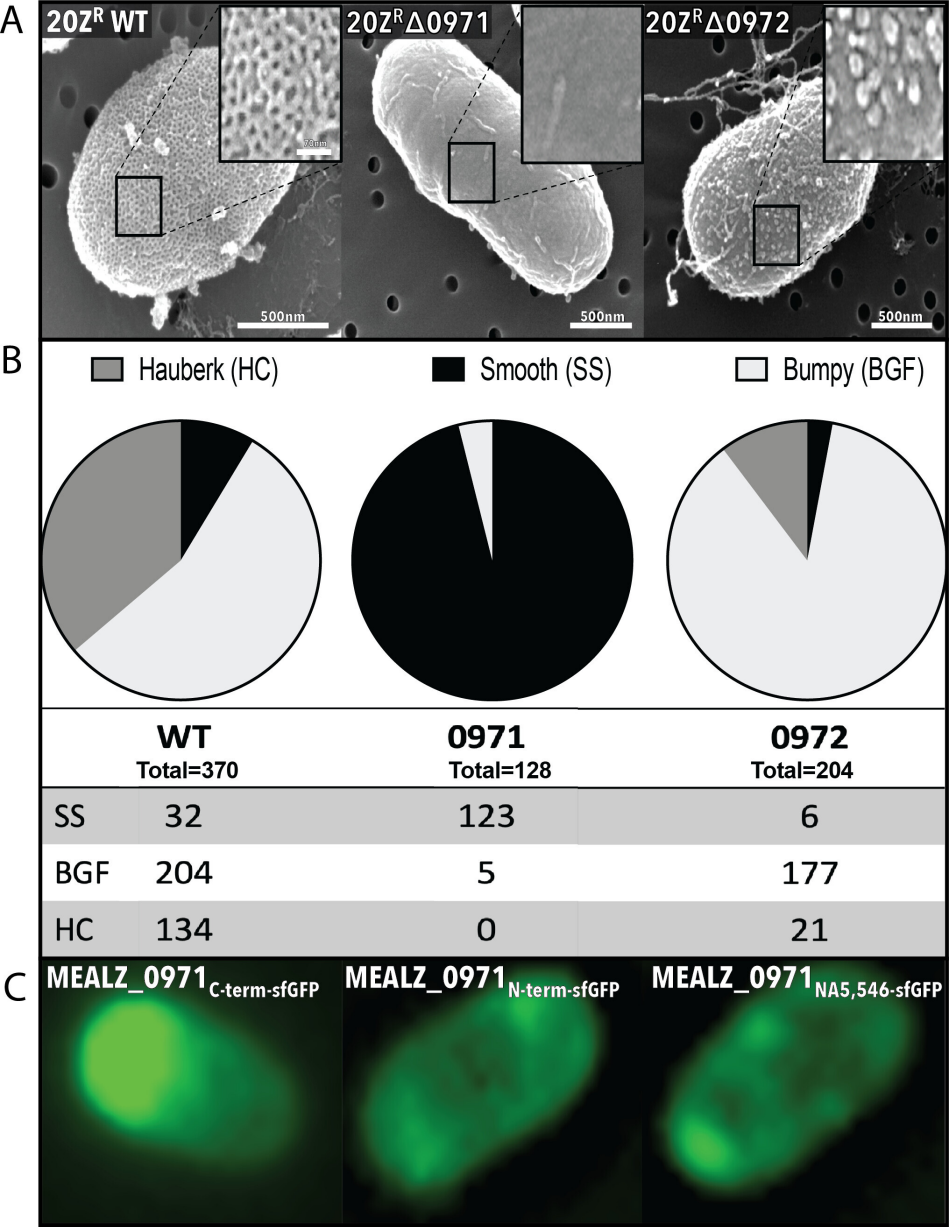


## A QUALITY READ OF MYCOBIOME METABARCODE SEQUENCES

Kyle and Klassen (e01537-24) warn about quality aberrations of untrimmed ITS2 metabarcode sequences that underestimate the relative abundances of specific fungal taxa. This is an important finding to ensure the correct description of mycobiomes.


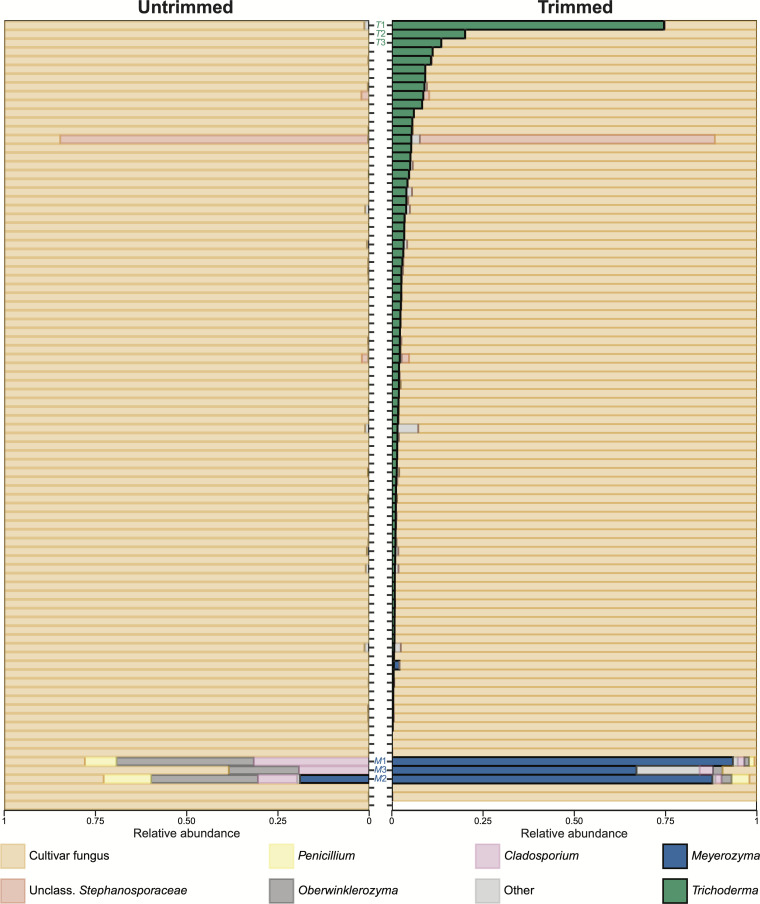


## APPLE ROOTSTOCKS MEET THEIR MYCORRHIZAL FUNGAL MATCH

Arbuscular mycorrhizal fungi (AMF) are promoted as commercial bioinoculants for sustainable agriculture. Zhang et al. (e01937-24) tested the stability of the bioinoculants in apple rootstocks. The results suggest that nursery-established AMF associations can be maintained when transplanted into the field but in a genotype-specific manner.


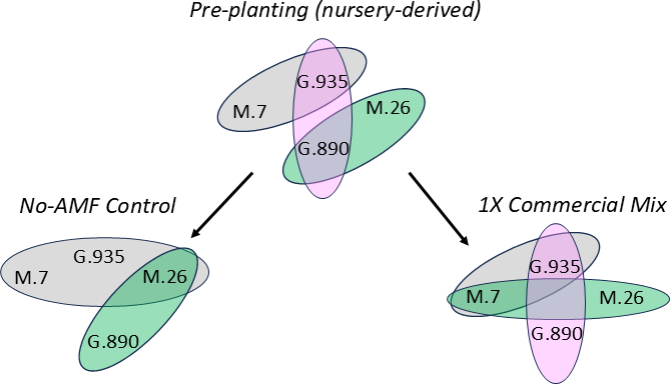


## SMALL BUT MIGHTY: A RHIZOBIAL HEAT SHOCK PROTEIN KEY TO NITROGEN-FIXING SYMBIOSIS WITH PLANTS

Rhizobia face numerous host-induced stresses during the establishment of their symbiosis with legume plants. Domingo-Serrano et al. (e01385-24) show that a small heat shock protein (sHSP) targets numerous proteins to protect essential bacteroid functions in the plant host and ensure optimal nitrogen-fixing symbiosis.


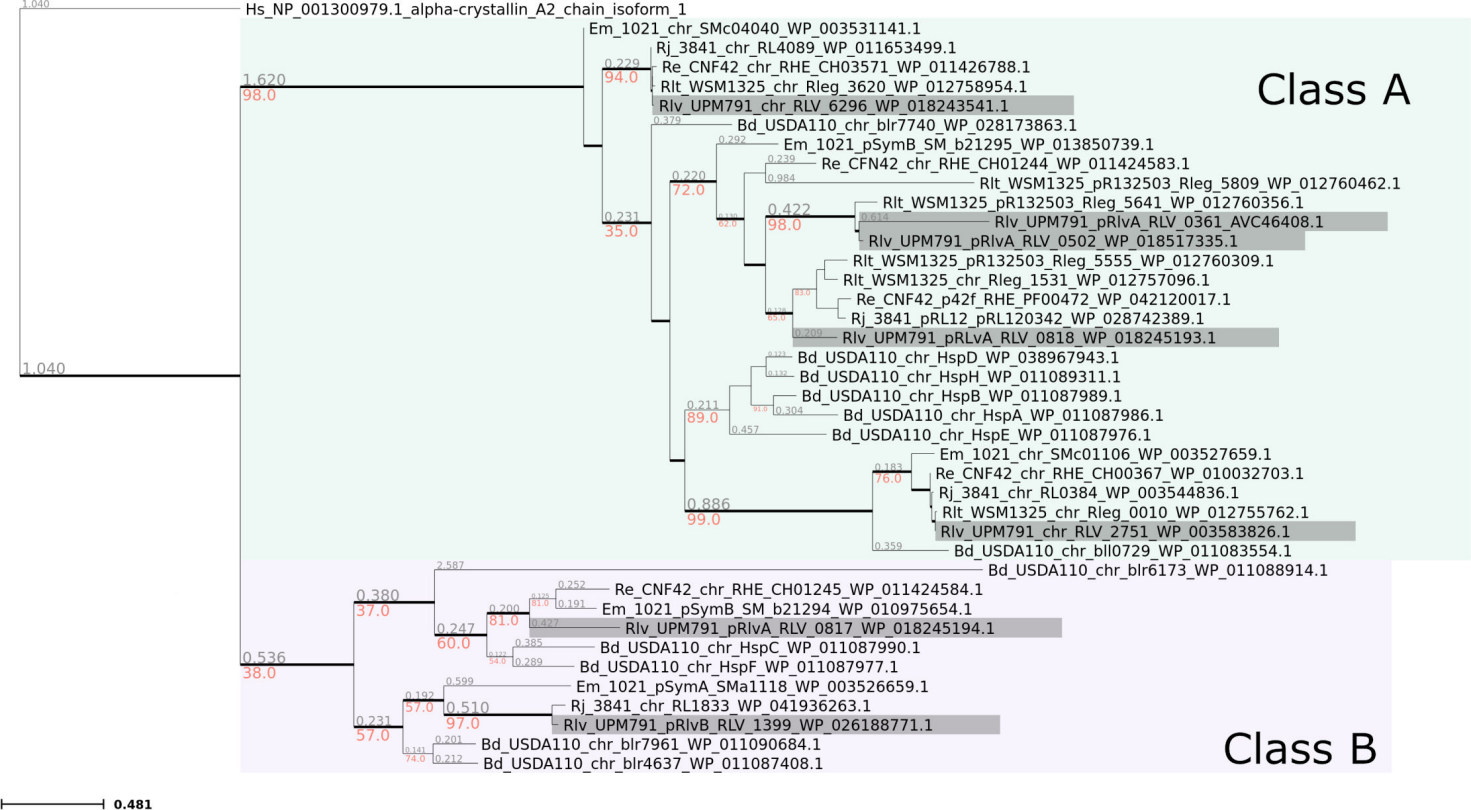


## TAPPING INTO THE NATURAL BIODIVERSITY OF YEASTS IN TROPICAL WEST AFRICA

A large bioprospecting study by Persson et al. (e01615-24) identified yeasts that efficiently converted lactose into lipids. These isolates show promise for the revalorization of the abundant side stream cheese whey from dairy industries.